# Fast and Cost-Effective Synthesis of High-Quality Graphene on Copper Foils Using High-Current Arc Evaporation

**DOI:** 10.3390/ma11050804

**Published:** 2018-05-16

**Authors:** Helge Lux, Matthias Edling, Peter Siemroth, Sigurd Schrader

**Affiliations:** 1 Department of Engineering and Nature Science, Technical University of Applied Sciences Wildau, Hochschulring 1, 15745 Wildau, Germany; edling@th-wildau.de (M.E.); schrader@th-wildau.de (S.S.); 2 Arc Precision, Sources, Coatings, and Analysis GmbH, Schwarzkopffstraße 2, 15745 Wildau, Germany; siemroth@arcprecision.com

**Keywords:** graphene, PVD, arc evaporation, copper, transfer, Φ-HCA

## Abstract

In this paper, we present an innovative and ultra-fast process for the deposition of high-quality graphene on different metal foils and thin metal films. The graphene layer can be homogeneously deposited in only 30 s process time. Due to the weak adhesion to the substrate material, the monolayer graphene is easy to transfer using the established processes. For the production, we use magnetic filtered high-current arc evaporation (Φ-HCA) with a solid, graphitic carbon source. This ultra-fast growth process can pave the way towards a cost-effective graphene synthesis for the mass production e.g., in a roll-to-roll process, avoiding time consuming established processes.

## 1. Introduction

Graphene, the monolayer modification of sp^2^-hybridized carbon in a honeycomb lattice, has attracted strong interest as a new material for microelectronics, photonics, sensors, and many other applications [1]. However, to introduce graphene into a broad industrial field, the deposition process must be stable, fast, and cost-effective to be suitable e.g., for a roll-to-roll technique.

So far, the most established method to fabricate large area monolayer graphene films is a CVD (chemical vapor deposition) or PE-CVD (Plasma enhanced CVD) process using carbon containing precursor gases like methane [2,3,4,5]. Using one of these processes, graphene grows on the surface of copper, nickel or other suitable metals with catalytic properties and exhibits high quality and cover large areas. Additionally, a roll-to-roll process using CVD was realized and published in 2010 [6,7]. However, most of the CVD and PE-CVD processes are time-consuming. The growth process of high quality graphene on multi crystalline copper foil lasts approximately 0.5 h, which is much too slow for an industrial process [3]. Consequently, the maximum reachable speed for producing high-quality closed graphene was reported to be 2 mm/s [7].

In contrast, arc evaporation as one of the most established physical vapor deposition methods (PVD) can produce a wide range of carbon nano-structures. Fullerenes, Nanotubes, and Carbon Black can be produced just by changing the deposition parameters, e.g., substrate temperature, gas composition, gas pressure, and plasma power [8,9]. Even the production of graphene nano-flakes by arc discharge in air or functional gases (hydrogen, helium) was recently published by diverse groups [10,11,12]. In this process, graphene nano-flakes have different thicknesses (few layer sheets) and cover the substrate randomly orientated without building a homogeneous monolayer of graphene. However, this kind of coatings can be interesting for several applications such as electrodes for batteries, energy storage systems, super caps, and so on.

If graphene should be used as a substitute for transparent conductive oxides (TCO) using the high transparency and excellent electrical conductivity or as a material for high frequency applications, a closed monolayer coating with low defects is necessary. These requirements cannot be fulfilled using the state-of-the-art arc evaporation processes as described above.

Therefore, we here present an innovative approach, which allows the ultra-fast growth of homogeneous large-area monolayer graphene (several cm^2^) on poly crystalline copper foils for 30 s. As far as we know, this is the first publication reporting the formation of low-defective closed graphene films using an arc evaporation method.

The basic idea of the process is to modify a magnetic filtered high current arc evaporation system to increase the sp^2^ content to form a closed graphene film instead of predominantly sp^3^-hybridized carbon.

In this paper, we used an arc evaporation tool which was originally developed to produce ultra-hard carbon films (ta-C, tetrahedral, amorphous carbon) with a high percentage of sp^3^ bonds [13,14]. In our new process, a reduction of the impinging energy of ions with inert and functional gases (argon, hydrogen) can avoid deep implantation into the substrate material and provides a gentle atmosphere to grow a single layer sp^2^-bound carbon instead of ta-C. Simultaneous heating of the substrate provides a limited mobility to the deposited atoms and should allow them to self-organize into a graphene layer. This modified deposition process is different from the well-known process of ex-post-heating (thermal annealing) of highly sp^3^-hybridized amorphous carbon films resulting in a predominantly sp^2^-hybridized graphite-like structure with a strongly reduced resistivity [15,16].

In contrast, in the process describe here, the carbon deposition and the thermal organization, resulting in a closed graphene film, occurs simultaneously. This deposition can be optimized for different substrate materials by several external parameters such as pressure and composition of background gas, temperature, impinging angle, and deposition rate. Using this technique, the deposition of transparent and conductive graphene-like carbon coatings on Insulators is also possible [17]. 

**Figure 1:**
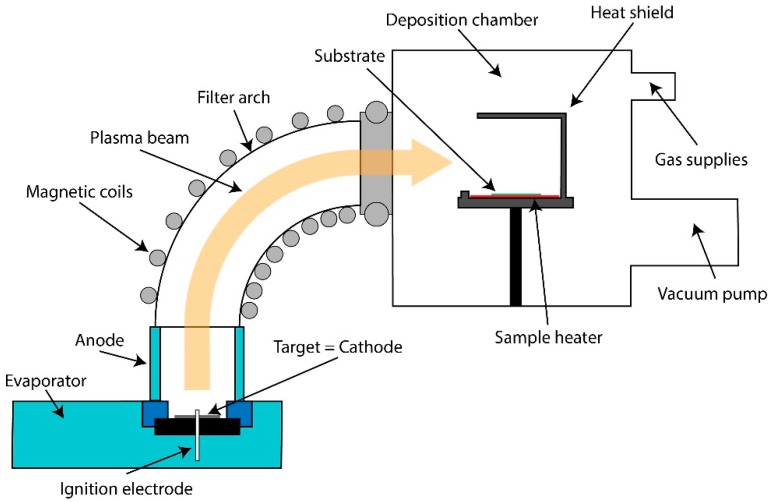
Filter arc setup for carbon deposition.

The fundamental design of the arc coating tool used in the present study is shown in Figure 1. The evaporator is connected to the deposition chamber by a magnetic particle filter. As carbon source, the filtered arc system (Φ-HCA) with a solid graphite target, described in more detail in [18], was used.

The evaporator works in a pulsed mode using 1.0 ms pulses with an arc current of up to 3000 A. For the current process only a small amount of carbon is necessary, corresponding to the atomically thin thickness of the graphene structure. Consequently, only 17 plasma pulses are necessary to form a closed graphene film. That fact makes the growth process extremely fast. After igniting the plasma discharge, the ions are firstly guided through the gas atmosphere in order to reduce their kinetic energy by collisions with the gas atoms. Additionally, hydrogen was used to reduce the crystal nucleus on the surface and enhance the growth of larger grains. The impinging angle of the carbon was changed in order to reduce the effective impact energy. The best conditions were obtained for an angle of incidence of 0° (grazing incidence, see Figure 1).

For the deposition of graphene instead of amorphous carbon, substrates must be heated before and during deposition. Therefore, we used a boron nitride heating plate, which is able to heat up the copper substrates near to the melting point.

## 2. Materials and Methods

As substrate material, we used 25 µm thin poly-crystalline copper foils (Alfa Aesar CAS-Number 7440-50-8). The copper foil was firstly cleaned in acetone and isopropyl alcohol and subsequently treated by chemical polishing using a mixture of acetic acid, nitric acid, and sulfuric acid [19] for 5 min in order to remove the macroscopic roughness of the copper for better graphene transfer.

Arc-Evaporation graphene was produced using the following process parameters: arc current 1500 A, magnetic filter current 200 A, pulse time 1 ms, pulse interval of 3 s, partial argon pressure 100 Pa, partial hydrogen pressure 20 Pa, substrate temperature 1020 °C. Before starting the coating process, the substrate was pre-heated for 20 min in 45 Pa hydrogen in order to increase the grain size of the copper foil and to remove the native oxide. After coating, the samples were cooled down for 10 min in argon/hydrogen atmosphere.

For comparison, CVD graphene was produced on copper using the following parameters: argon flow 90 sccm, hydrogen flow 20 sccm, methane flow 20 sccm, overall working pressure 70 Pa coating time 30 min, substrate temperature 1000 °C. Before starting the coating process, the substrate was pre-heated for 30 min with 60 sccm argon in order to increase the grain size of the copper foil and to remove the native oxide. After coating, the samples were cooled down for 20 min in argon atmosphere.

For transfer, an established electrolytic transfer process was used [20,21]: 1 M NaOH in water, voltage 2.7 V, transport layer 1 µm PMMA (350 k, spin coated). After dissolution from the copper, the graphene–polymer stack was three times rinsed in highly pure water and subsequently fished out by using the target material (300 nm SiO_2_ on Silicon) followed by drying in atmosphere and baking procedure (5 min at 80 °C, 5 min 120 °C). For removing the PMMA, the sample was washed in Acetone for 20 min and subsequently rinsed in Isopropyl alcohol and pure water.

Raman measurements were conducted using a WiTec alpha 300 micro Raman microscope using 488 nm excitation laser wavelength with 30 mW with a lens magnification of 50×. The single spectra on one point were taken out from the large area scan with an integration time of 100 ms and one accumulation. Mappings were made with a lateral resolution of 0.66 µm per pixel with an integration time of 100 ms. For comparison for each of these mappings an average spectrum from every point inside the scanned area was created.

## 3. Results

### 3.1. Parameter Optimization

As described above, the deposition process can be carefully optimized by changing the deposition parameters. The amount of carbon which is deposited on the substrate surface is determined by the number of plasma pulses. Consequently, this parameter is the most significant one to form a monolayer of graphene, because arc evaporation is not self-limiting. For the current study, we varied the number of plasma pulses from 10 to 35 with an arc current of 2.5 kA to identify the best conditions for monolayer graphene. After the deposition, we characterized the graphene using high-resolution Raman mapping. For all samples, we observed the typical Raman spectrum for graphene with the characteristic D-peak at 1350 cm^−1^, G-peak at 1581 cm^−1^, and 2D-peak at 2703 cm^−1^. The D-peak, originating from defects or grain boundaries which are breaking the translational symmetry of the Raman scattering process [22]. For monolayer graphene, the Raman I(2D)/I(G) ratio reaches values higher than two and decreases by an increasing number of layers. Additionally, the peak position if the 2D peak shifts to higher wavenumbers. Consequently, the height and position of the 2D peak strongly depends on the number of graphene layers.

**Figure 2:**
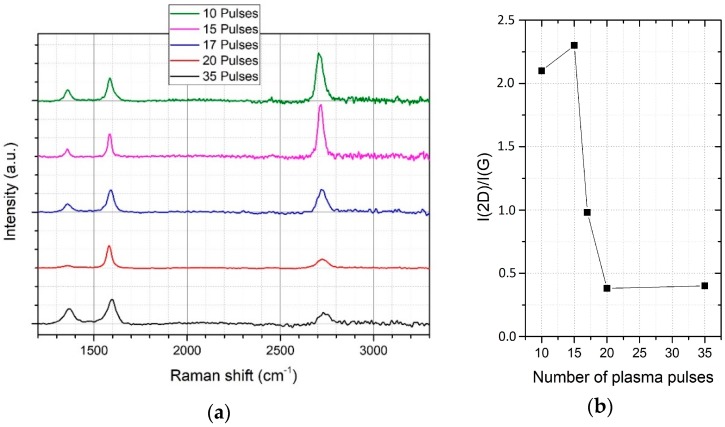
(**a**) Average Raman spectrum from the large area scan for a different number of pulses, (**b**) Raman I(2D)/I(G) ratio vs. number of pulses.

Figure 2a shows the average Raman spectrum from the large area scan for a different number of pulses, Figure 2b the change in I(2D)/I(G) ratio.

After 10 plasma pulses, the graphene growth is still incomplete, resulting in a beginning nucleation of graphene domains and uncoated regions. This can be seen by a decreasing Raman I(2D)/I(G) ratio compared to 15 plasma pulses. After 15 pulses, we recognized a complete coverage of monolayer graphene. By increasing the number of plasma pulses, the Raman I(2D)/I(G) ratio decreases. Additionally, the 2D-peak position shifts to higher wave numbers, indicating the growth of multilayer graphene, as reported in [22]. After 35 pulses, the D-peak strongly increases, indicating an amorphous carbon growth on top of a closes graphene film. 

**Figure 3:**
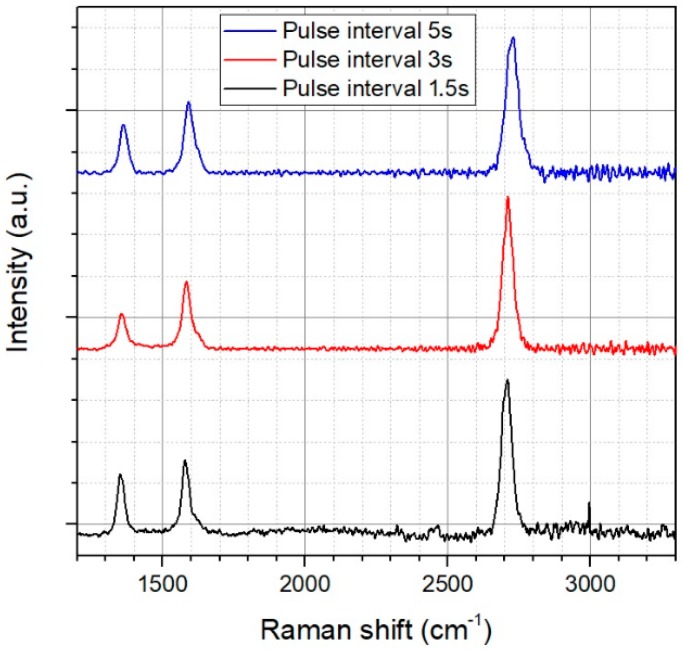
Average Raman spectrum from the large area scan for different time interval.

In order to speed up the process, we additionally varied the time between two plasma pulses (pulse interval) from 1.5 to 5 s. The shorter the pulse interval can be chosen, the higher is the effective deposition rate. Figure 3 shows the resulting Raman spectra of this time optimization process. In case of 1.5 s pulse interval, a high D-peak can be observed. This can be caused either by a too fast growth mechanism or by the residual gas, which is coming from the solid graphite target. If the time between two pulses is too short, this gas can accumulate inside the vacuum chamber and subsequently disturb the graphene growth. If the time between two plasma pulses rises above 5 s, background gas can be adsorbed on the surface of the copper substrate which results in defective graphene showing an increasing D-peak in Raman spectrum. For the time optimization, we identified a time interval of 3 s as optimum. By using 15 plasma pulses for growing a closed graphene film, the whole deposition process time is only 45 s. In the current chamber we are able to homogeneously coat an area of 100 × 100 cm^2^. Because the graphene growth ultimately limits the production rate [7], it would be possible to reach a production speed of 133 mm/s by integrating this technology in a standard roll-to-roll process. The copper foil itself can be heated up much faster, reaching a speed up to 5 m/min [7].

However, even by using an optimized pulse interval of 3 s, a non-neglectable D-peak can be seen, which is a sign of defective graphene.

**Figure 4:**
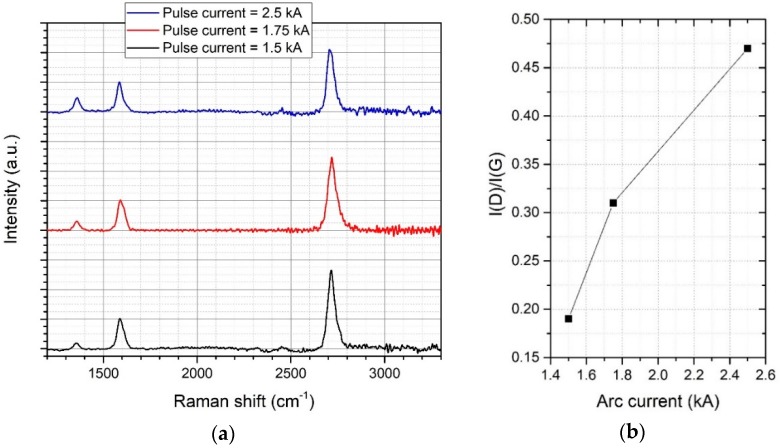
(**a**) Average Raman spectrum from the large area scan for different arc current, (**b**) Raman I(D)/I(G) ratio vs. arc current.

Consequently, for a further optimization of the growth process, we varied the plasma pulse current from 1.5 to 2.5 kA. The plasma pulse current determines the plasma density during the deposition. Hence, this parameter influences the deposition rate of the growing film and subsequently the number of defects and the grain size of the graphene layer. Figure 4a shows the average Raman spectrum from the large area scan for different arc current, Figure 4b the change in Raman I(D)/I(G) ratio. It can clearly be seen that the D-peak significantly decreases by decreasing the plasma current, indicating a lower defect density and higher quality of the graphene. In contrast, the Raman I(2D)/I(G) ratio is constant and shows the presence of monolayer graphene.

### 3.2. Results for Optimized Parameters

**Figure 5:**
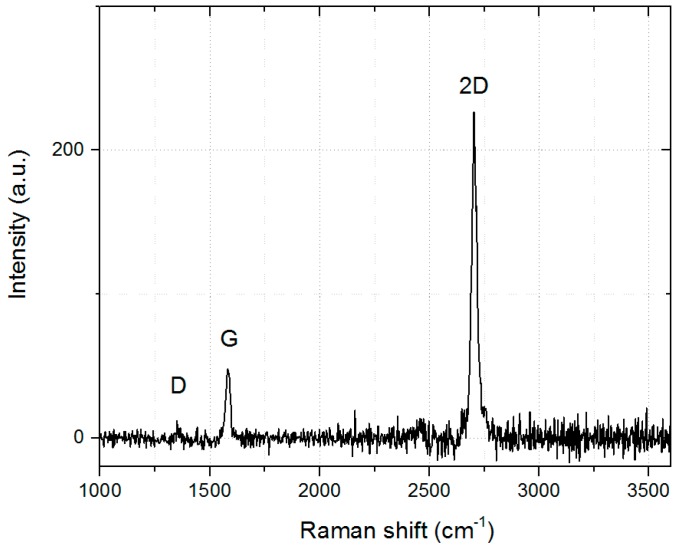
Single Raman spectrum of the as-grown graphene on copper.

Using the previously described optimized parameters of the modified Φ-HCA process, it is possible to reproducibly deposit high quality graphene on poly-crystalline copper foils. Figure 5 shows a single Raman spectrum which was taken out from a large area surface scan.

The 2D band is roughly 4 times higher than the G peak (Graphite) which is a sign of single layer graphene with only few defects [22]. Additionally, the 2D band is sharp, having a full width at half maximum (FWHM) of only 28 cm^−1^. The D-band, originating from defects or grain boundaries which are breaking the translational symmetry of the Raman scattering process [22], is almost negligible. Main Raman peak properties are summarized in Table 1.

**Figure 6:**
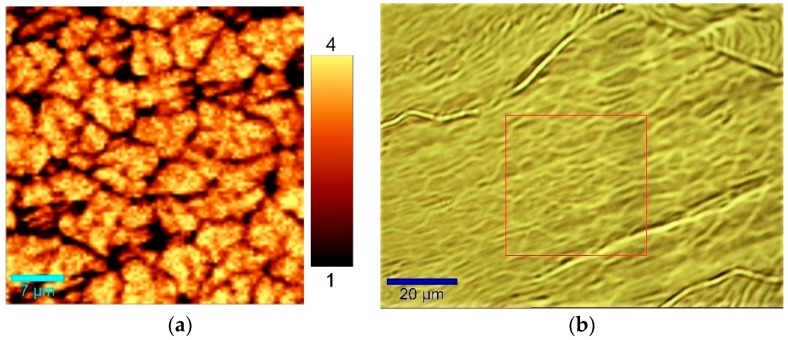
(**a**) Raman mapping of the Raman I(2D)/I(G) ratio; (**b**) Microscopic image of the copper surface after graphene deposition.

For investigating the homogeneity of the graphene layer, large area Raman scans with high resolution were conducted. Figure 6a shows the I(2D)/I(G) ratio on a surface area of 2500 µm^2^. Figure 6b contains the microscopic image with the marked region for Raman mapping (red rectangle).

The Raman I(2D)/I(G) ratio is predominantly above 3 inside the graphene grains, which clearly shows the formation of high-quality single layer graphene. Variation in Raman I(2D)/I(G) intensity was created by the grain boundaries of the single layer graphene. Surprisingly, the graphene layer can be identified even in the microscopic image of the copper surface (Figure 6b), indicating a grain size of about 10 µm. 

**Figure 7:**
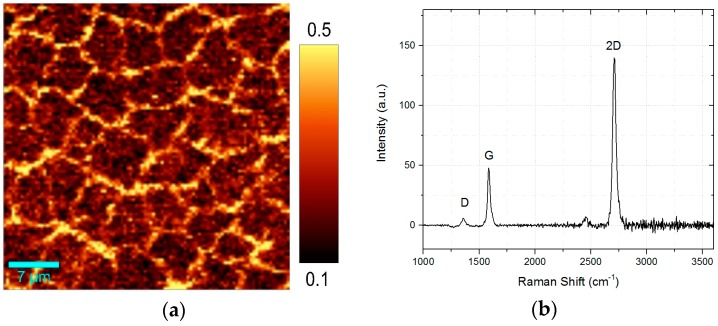
(**a**) Raman mapping of the Raman I(D)/I(G) ratio; (**b**) Average Raman spectrum of 2500 µm^2^ graphene area.

Furthermore, Figure 7a shows a high-resolution Raman I(D)/I(G) mapping. As mentioned before, the grain boundaries can be observed by a much higher D-band. Consequently, in the average Raman spectrum of the analyzed area, a small D-band is visible (Figure 7b).

Subsequently, the graphene layer was transferred for further investigation on oxidized silicon (300 nm thermal grown SiO_2_ on Silicon wafer) using the established electro-chemical transfer [21].

### 3.3. Transfer and Comparison to CVD-Graphene

**Figure 8:**
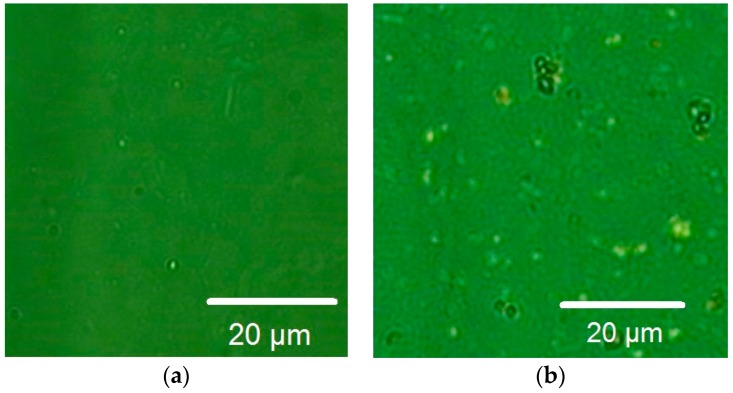
(**a**) Microscopic images of transferred graphene on 300 nm SiO_2_ on Silicon: (**a**) Arc-Evaporation-graphene, (**b**) CVD-grown graphene.

To compare our Φ-HCA-graphene with graphene grown on copper foils using CVD, we additionally prepared samples by means of a standard CVD process (for more detail, see Section 2). Then, we transferred both graphene layers on 300 nm SiO_2_ on Silicon using the same electro-chemical method. Figure 8 shows microscopic images of the transferred graphene layers on 300 nm SiO_2_ on Silicon: (a) Φ-HCA-graphene, (b) CVD-graphene.

The transferred CVD-graphene shows the typical residuals of the transport polymer and the substrate material (copper). In contrast, the Arc-Evaporation-graphene is almost free of copper residuals and shows only few contaminations with the polymer film. Additionally, the Arc-Evaporation-graphene was much easier to transfer. It seems that the adhesion between the Arc-graphene and the copper is much weaker than in case of transferring standard CVD-graphene. This can be caused by a completely different growth process compared to CVD.

**Figure 9:**
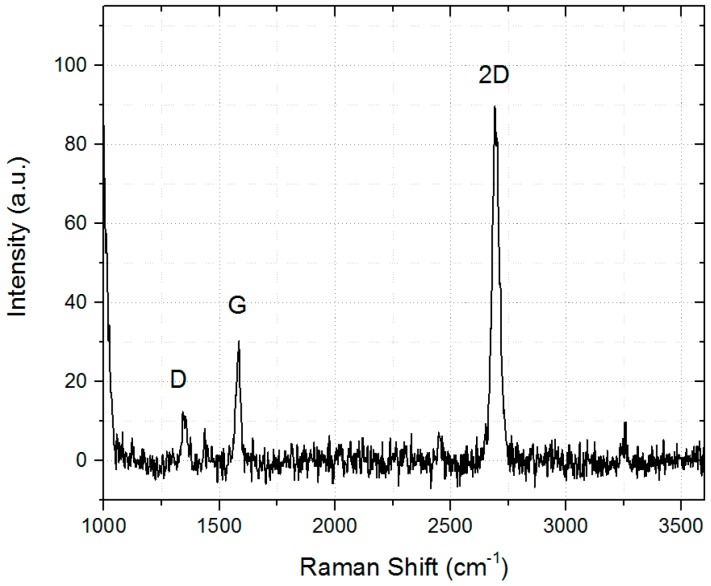
Raman single spectrum of the transferred Arc-graphene on 300 nm Silicon dioxide on Silicon using electro-chemical transfer.

Figure 9 shows a single Raman spectrum of the transferred Arc-graphene on 300 nm Silicon dioxide on Silicon using electro-chemical transfer. 

The Raman I(2D)/I(G) reaches the value of 3 indicating the presence of monolayer graphene whereas the intensity of the D-band increases. The presence of D-band requires defects (caused by mechanical stress, cracks, folds, amorphous carbon, grain boundaries), which can be a result of the treatment during the transfer process [20]. Hence, the D-band is not a result from our coating process but can be decreased by optimizing the transfer.

**Figure 10:**
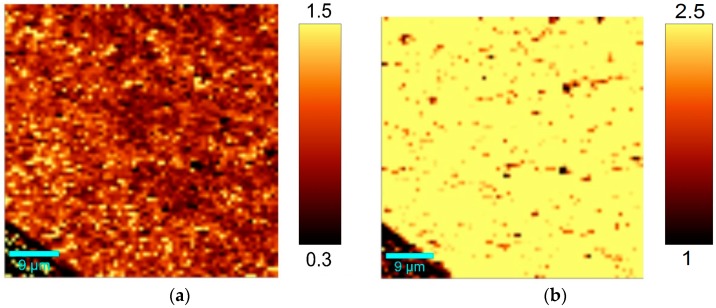
(**a**) Raman mapping of I(D)/I(G) ratio, (**b**) Raman mapping of I(2D)/I(G) ratio.

The Raman I(D)/I(G) ratio (Figure 10a) as well as the Raman I(2D)/I(G) ratio (Figure 10b) is homogeneous distributed over the scanning area without any visible holes. The dark regions in the I(2D)/I(G) mapping indicate a decreasing 2D band, caused by multi-layer domains, impurities of the substrate material or defective graphene. The edge in the bottom left corner marks the transition to the uncoated substrate material (edge of the graphene layer).

Additionally, electrical properties of the transferred graphene on Silicon oxide were characterized by means of four tip measurements (van der Pauw arrangement) and Hall measurement. Due to some defects after the transfer process, our transferred graphene reaches a sheet resistance of 4 kΩ/square and a charge carrier mobility of 400 cm^2^/Vs for one monolayer. The electrical properties can be further enhanced by using an optimized transfer process. However, these values reported here are already comparable to other established methods [23,24].

Overall, a monolayer graphene with only few defects can be grown very fast on multi crystalline copper foils using this new Φ-HCA process and subsequently transferred by means of electro-chemical methods.

## 4. Conclusions

A new ultra-fast deposition process for graphene on copper foils is presented. A modified magnetic filtered vacuum arc system (Φ-HCA) with hydrogen containing argon as background gas and with heated and tilted substrates can form graphene with excellent homogeneity covering large areas. The filtered arc source supplies the surface only with that amount of material which is necessary for the reorganization of carbon atoms into graphene. Consequently, only 17 plasma pulses are necessary to form a closes graphene film. This makes the deposition process much faster than many established CVD processes.

After transfer, the graphene shows almost negligible copper residuals and only few contaminations of the polymer transportation layer.

To sum up, this fast and cost-effective process can pave the way towards an industrial roll-to-roll process and opens a broad field of application of this kind of carbon layers.

## Figures and Tables

**Table 1:** Peak properties of Raman spectrum of graphene on copper.

Raman Peak	Max. Position	FWHM
G	1581 cm^−1^	24 cm^−1^
2D	2703 cm^−1^	28 cm^−1^
